# Alzheimer's pathology in primary progressive aphasia

**DOI:** 10.1016/j.neurobiolaging.2010.05.020

**Published:** 2012-04

**Authors:** Jonathan D. Rohrer, Martin N. Rossor, Jason D. Warren

**Affiliations:** Dementia Research Centre, Department of Neurodegenerative Disease, UCL Institute of Neurology, University College London, Queen Square, London, United Kingdom

**Keywords:** Frontotemporal dementia, Frontotemporal lobar degeneration, Primary progressive aphasia, Logopenic aphasia, Progressive nonfluent aphasia, Alzheimer's disease

## Abstract

Primary progressive aphasia (PPA) is a neurodegenerative disorder with language impairment as the primary feature. Different subtypes have been described and the 3 best characterized are progressive nonfluent aphasia (PNFA), semantic dementia (SD) and logopenic/phonological aphasia (LPA). Of these subtypes, LPA is most commonly associated with Alzheimer's disease (AD) pathology. However, the features of PPA associated with AD have not been fully defined. Here we retrospectively identified 14 patients with PPA and either pathologically confirmed AD or cerebrospinal fluid (CSF) biomarkers consistent with AD. Analysis of neurological and neuropsychological features revealed that all patients had a syndrome of LPA with relatively nonfluent spontaneous speech, phonemic errors, and reduced digit span; most patients also had impaired verbal episodic memory. Analysis of the pattern of cortical thinning in these patients revealed left posterior superior temporal, inferior parietal, medial temporal, and posterior cingulate involvement and in patients with more severe disease, increasing involvement of left anterior temporal and frontal cortices and right hemisphere areas in the temporo-parietal junction, posterior cingulate, and medial temporal lobe. We propose that LPA may be a “unihemispheric” presentation of AD, and discuss this concept in relation to accumulating evidence concerning language dysfunction in AD.

## 1 Introduction

Primary progressive aphasia (PPA) refers to a group of neurodegenerative disorders with language impairment as the initial symptom (Mesulam, 1982, 2001, 2003). These disorders are of high neurobiological and clinical importance because they illustrate the potentially focal nature of neurodegenerative disease and the potential heterogeneity of clinical presentations even where the underlying pathological process is uniform. The best characterized subtypes of PPA are progressive nonfluent aphasia (PNFA) and semantic dementia (SD). Patients with PNFA have nonfluent speech characterized by agrammatism and/or a motor speech impairment (usually an apraxia of speech, i.e., hesitancy and effortfulness attributable to impaired planning of articulation) (Ogar et al., 2007). SD presents with fluent aphasia, anomia, and single word comprehension deficits secondary to verbal semantic impairment (Hodges and Patterson, 2007). “Fluency” in this context refers to the flow of speech. However, dysfluency may arise from a variety of underlying deficits, including agrammatism, impaired articulation (motor deficits such as apraxia of speech), decreased phrase length or slower speech rate (e.g., due to word-finding pauses); patients referred to as having a “nonfluent aphasia” may have various more or less distinct primary language or speech impairments. This theme is well illustrated by the recently recognized entity of logopenic/phonological aphasia (LPA) (Gorno-Tempini et al., 2004, 2008), which constitutes a third major syndrome within the PPA spectrum. Patients with LPA have word-finding pauses and anomia as well as impaired speech repetition, particularly sentences (Gorno-Tempini et al., 2008).

Most cases of PPA have a non-Alzheimer pathological substrate within the frontotemporal lobar degeneration spectrum, and are usually associated predominantly with either tau- or TAR (trans-activation-response) DNA binding protein 43 (TDP-43)-positive cellular inclusions (known as FTLD-tau or FTLD-TDP pathology), respectively (Knibb et al., 2006; Snowden et al., 2007). However, it has long been recognized that PPA syndromes may also be associated with Alzheimer's disease (AD) pathology (Clark et al., 2003; Green et al., 1990, 1996; Karbe et al., 1993; Kempler et al., 1990; Li et al., 2000; Pogacar and Williams, 1984) and in recent years more detailed series have been reported (Alladi et al., 2007; Croot et al., 2000; Davies et al., 2005; Galton et al., 2000; Josephs et al., 2008; Kertesz et al., 2005; Knibb et al., 2006; Mesulam et al., 2008). In particular, recent evidence has suggested that LPA is underpinned by AD pathology in a high proportion of cases and may be the most common aphasia phenotype of AD (Gorno-Tempini et al., 2008; Mesulam et al., 2008; Rabinovici et al., 2008). However both PNFA and SD have also been reported with AD pathology, as have syndromes that do not fit clearly into a single category, so-called “mixed” aphasia (Alladi et al., 2007; Knibb et al., 2006). As AD is the most common neurodegenerative disease of later life, the range of phenotypic variation in AD and the mechanisms that drive this variation are key issues in the field of neurodegenerative disease.

Here we review the clinical, neuropsychological and cross-sectional neuroimaging features of a retrospective series of patients with a clinical diagnosis of PPA and AD pathology either demonstrated directly or presumed on the basis of cerebrospinal fluid (CSF) biomarker profiles. We consider these cases in relation to previously published series of PPA patients with either pathologically confirmed AD or a positive Pittsburgh compound B (PIB)-positron emission tomography (PET) scan suggestive of AD.

## 2 Methods

From the Dementia Research Centre patient database comprising a consecutive series of patients seen between 1992 and 2008, we extracted all cases meeting criteria for PPA (Mesulam, 2001, 2003) and who had either AD pathology at postmortem/cerebral biopsy or CSF biomarker data consistent with Alzheimer pathology (raised CSF total tau level with reduced amyloid Aβ42 fraction; Blennow and Hampel, 2003; Hulstaert et al., 1999; Tapiola et al., 2009). In total, 14 patients were included in the series: 9 had pathologically confirmed Alzheimer's disease (7 who came to postmortem and 2 on cerebral biopsy) and 5 had CSF biomarkers consistent with AD (these 5 patients were previously reported in Rohrer et al., 2010). Clinical notes and neuropsychological data were reviewed, and the clinical diagnosis at the time the patient was initially assessed and a revised clinical diagnosis based on current descriptive criteria for PPA (Mesulam, 2001, 2003; Gorno-Tempini et al., 2004, 2008) were recorded in each case. Neuropsychological data were also recorded where available. Ethical approval for the study was obtained from the National Hospital for Neurology and Neurosurgery Local Research Ethics Committee. Written research consent was obtained from all patients participating in the study.

### 2.1 Brain imaging analysis

All subjects had been scanned on a 1.5 T GE Signa unit scanner (General Electric, Milwaukee, WI) with T1-weighted volumetric images obtained with a 24-cm field of view and 256 × 256 matrix to provide 124 contiguous 1.5-mm-thick slices in the coronal plane. Mean (standard deviation) age at scan was 60.2 (6.2) years. A control group of 23 age- and gender-matched cognitively normal subjects (mean age 63.5 [7.3] years at time of scan) was used for comparison. No subject had significant cerebrovascular disease or other secondary pathology on neuroimaging. Image analysis was performed using the MIDAS software package (Freeborough et al., 1997). A rapid, semiautomated technique of brain segmentation which involves interactive selection of thresholds, followed by a series of erosions and dilations was performed for each scan. This yielded a brain region which was separated from surrounding CSF, skull, and dura giving a baseline brain volume. Ventricles were also segmented within MIDAS. Scans and associated brain regions were initially transformed into standard space by registration to the Montreal Neurological Institute (MNI) Template (Mazziotta et al., 1995). Left and right hemispheric regions were defined using the MNI average brain which was split by dividing the whole volume along a line coincident with the interhemispheric fissure. An intersection of each individual's brain region and the hemispheric regions defined on the MNI template was generated to provide a measure of brain volume in left and right hemispheres and left/right volume ratios were also calculated. The 2 disease groups and the healthy control group were compared statistically based on contrasts between the group means using a linear regression model in STATA10 (Stata Corporation, College Station, TX).

We investigated changes in imaging patterns with severity using cortical reconstruction and thickness estimation methods with the Freesurfer image analysis suite (surfer.nmr.mgh.harvard.edu/) as previously described (Rohrer et al., 2009). We used performance on the Graded Naming Test (McKenna and Warrington, 1980, total number of items equals 30) (i.e., degree of anomia) as a measure of disease severity, splitting the group according to their score: group 1 (less severe: 9 patients) scored > 0 (mean 7.7, standard deviation 9.2) and group 2 (more severe: 4 patients) were unable to score. One case (AD-PPA6) with greater right than left hemisphere atrophy was not included in this analysis; this atrophy profile might reflect either a different disease phenotype or reversed hemisphere language dominance, however inclusion of this case could potentially bias any group-level correlations between cortical thickness and disease severity.

Effect size maps were generated based on the difference in mean thickness in each of these severity subgroups and in the whole group, comparing each to the controls and expressing the disease-control difference as a percentage of the mean control group thickness.

## 3 Results

### 3.1 Clinical and neuropsychological features

Demographic and clinical data for patients are presented in Table 1; neuropsychological data (where available) are presented in Table 2. All patients had language impairment as their primary presenting feature. This was usually difficulty finding words although 1 patient complained of a return of a childhood stutter shortly before the onset of word-finding difficulties. Spontaneous speech was relatively nonfluent and occasional phonemic errors were made by all patients, with occasional emergence of neologistic jargon errors. None of the patients was described as having had apraxia of speech. All of the patients who came to postmortem or had a cerebral biopsy had initially received a diagnosis of PPA, PNFA, or language variant frontotemporal lobar degeneration although prior to death the diagnosis in 2 of these cases was changed to atypical language variant of AD. The 5 patients with CSF biomarkers consistent with AD were ascertained more recently and had been diagnosed with LPA before CSF analysis. On review of the clinical notes of the 7 patients who came to postmortem and the 2 patients with cerebral biopsy-proven AD, all would also have met criteria for LPA based on their initial symptoms and neurocognitive assessment. A family history of dementia was present in only 2 cases: these patients each had a single parent with a diagnosis of Alzheimer's disease in the eighth decade. Myoclonus was noted in 2 patients and 2 patients developed generalized seizures. One patient exhibited axial rigidity late in the course of the disease; no other features of parkinsonism or motor neuron disease were present in this series. Behavioral impairment was unusual early in the illness but aggression, anxiety, and irritability were noted in some patients later in the course.

Although all patients had had an initial neurocognitive assessment, for many patients formal neuropsychological testing was only performed later in the illness (e.g., when AD-PPA4 was tested, Mini Mental State Examination [MMSE] score was 4/30 and he performed poorly across multiple domains). Consistent with a diagnosis of LPA, neuropsychological assessment showed severely impaired digit span in all but 3 patients, who scored in the low (but not defective) range. Naming was in the impaired range at initial assessment in over half of the patients and became impaired in all cases as the disease progressed, also consistent with LPA. Single-word comprehension was intact in 9 of 14 patients as has been described in LPA but impaired in the more severely affected patients (intact in those with MMSE 18 or above, impaired in those with an MMSE below 17). None of the patients complained of episodic memory impairment at presentation, however verbal memory was impaired in 8 of 11 patients tested while visual memory was affected less frequently (5 of 14 patients). Reading was affected in most patients and some were noted to have phonological dyslexia. Limb apraxia and dyscalculia were noted in most patients, however visuospatial skills were intact in all but 1 severely affected patient. Executive dysfunction was also seen in most patients.

### 3.2 Pathological features

Six of the 7 patients who came to postmortem had severe Alzheimer pathology with Braak stage VI, and Consortium to Establish a Registry for Alzheimer's Disease (CERAD) frequent plaques (Table 1). For the seventh case, no staging information was available but this case had been reported as showing severe Alzheimer pathology with frequent plaques and tangles. Four cases were also noted to have cerebral amyloid angiopathy. The 2 patients who had cerebral biopsies were noted to have frequent amyloid plaques and neurofibrillary tangles.

### 3.3 Neuroimaging features

**Fig. 1:**
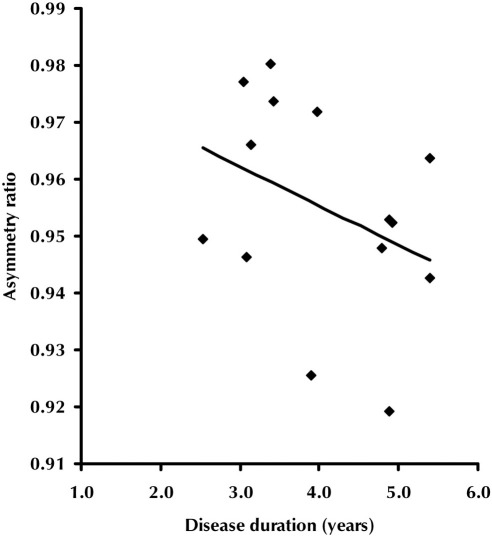
Asymmetry ratio (left/right hemisphere volumes) as a function of disease duration in years (based on cross-sectional data).

Volumetric magnetic resonance imaging (MRI) data for patients and controls are presented in Table 3. Whole brain and hemisphere volumes were smaller in patients and there was evidence of left/right hemispheric asymmetry at group level and in all but 1 of the individual patients; 1 (right-handed) patient showed reverse asymmetry. Asymmetry became more marked with increasing disease duration (Fig. 1, *R* = 0.55, *p* = 0.04).

**Fig. 2:**
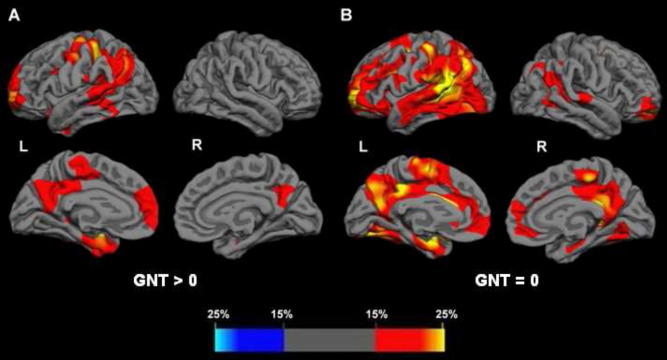
Patterns of cortical thinning in the Alzheimer's disease (AD)-primary progressive aphasia (PPA) groups versus healthy controls, categorized by severity of anomia: group 1, less severe (A); group 2, most severe (B). For each hemisphere, the top panels are lateral views, the bottom panels medial views. Percentage thinning maps are shown; the colored bar represents percentage values.

In the cortical thickness analysis versus healthy controls (Fig. 2), group 1 (with less severe disease) showed areas of cortical thinning predominantly in the left hemisphere, most marked in the inferior parietal and posterior superior temporal lobes. Other areas involved in the left hemisphere were posterior cingulate, precuneus, medial temporal lobe, and prefrontal cortex. In the right hemisphere, only the posterior cingulate and precuneus and a small area in the medial temporal lobe were affected. In group 2 with more severe anomia, cortical thinning remained asymmetrical but was more extensive within both hemispheres. In the left hemisphere there was additional involvement of anterior superior and middle temporal lobe, posterior medial temporal lobe, and inferior frontal lobe areas. In the right hemisphere there was involvement of areas similar to those initially involved in the left hemisphere, i.e., lateral parietal, posterior superior temporal, posterior cingulate, precuneus, medial temporal, and prefrontal cortices.

## 4 Discussion

Here we have described a series of 14 patients with PPA in association with proven or probable AD pathology. The key clinical features of the cases in this series were initial presentation with word-finding difficulty, and relatively nonfluent spontaneous speech with occasional phonemic errors but without motor speech impairment. “Word-finding difficulty”, like fluency, refers to a cluster of related deficits (Rohrer et al., 2008): though often related to anomia, patients with conversational pauses but with relatively intact naming may also present with a word-finding problem. Reviewing the diagnoses in this series revealed that all cases fulfilled (or would likely have fulfilled) descriptive criteria for LPA (Gorno-Tempini et al., 2004, 2008). The neuropsychological findings of impaired digit span, dyscalculia, limb apraxia, and phonological dyslexia were consistent with LPA (Amici et al., 2006; Brambati et al., 2009). However, verbal memory, although not a presenting feature in any of the patients, was also affected in most cases. Although this feature has not been emphasized in some previous studies of LPA, in 1 previous series 5 of 6 patients were impaired on verbal memory tasks (Gorno-Tempini et al., 2008). In contrast, visuospatial processing (a right hemisphere function) was generally well preserved. Cross-sectional brain imaging revealed asymmetrical left-sided atrophy predominantly affecting the posterior superior temporal lobe and inferior parietal lobe but also the posterior cingulate, precuneus, and medial temporal lobe. These features corroborate previous neuroanatomical findings in LPA (Gorno-Tempini et al., 2004, 2008). In more severe disease there was evidence of atrophy spread to the left frontal lobe, more anterior left temporal lobe areas, as well as posterior superior temporal lobe, inferior parietal lobe, and posterior cingulate areas within the right hemisphere.

The nosology of patients with language impairment and AD pathology remains controversial. Such patients have been classified either as having the symptomatic description of PPA (with an LPA phenotype in most cases) or having the predictive clinicopathological description of an atypical “language variant” within the AD spectrum. While there should not be conflict between these 2 descriptions as they are essentially at 2 different levels of classification, predicting which patients with a PPA syndrome will have AD pathology (particularly in the absence of a PIB-PET scan or CSF markers) is nevertheless often challenging during life. The extent of involvement of other cognitive domains may be helpful, however the present evidence suggests that the presence and severity of extralinguistic impairments depends on disease stage. Furthermore, the clinical salience of these additional impairments is variable: in this series, a number of patients that performed poorly on episodic memory tasks did not complain of amnestic symptoms, whereas 2 patients who came to postmortem exhibited widespread cognitive impairment prompting a reformulation of the clinical diagnosis as an atypical language variant of AD. We would argue that the presenting syndrome at an early disease stage is likely to provide the more rational basis for classifying language dysfunction associated with AD, particularly as language impairments are very common as “typical” AD advances. This distinction is clinically important, as recognition of PPA features that predict AD pathology could help direct the use of investigations such as CSF and PIB-PET, and ultimately, the selection of patients for clinical trials and disease-modifying therapies.

Previous series from 5 research groups have reported PPA patients with either pathologically confirmed AD or a positive PIB-PET scan showing amyloid deposition (Table 4; Alladi et al., 2007; Croot et al., 2000; Davies et al., 2005; Galton et al., 2000; Gorno-Tempini et al., 2008; Josephs et al., 2008; Kertesz et al., 2005; Knibb et al., 2006; Mesulam et al., 2008; Migliaccio et al., 2009; Pereira et al., 2009; Rabinovici et al., 2008). Prior to the first detailed description of LPA (Gorno-Tempini et al., 2004), patients with both PNFA and SD were reported with AD pathology but since that time LPA has been the clinical syndrome most closely associated with AD pathology. In Rabinovici et al. (2008), all patients with LPA versus 1 of 6 patients with PNFA and 1 of 5 patients with SD had positive PIB-PET scans; in Mesulam et al. (2008), 7 of 11 logopenic patients had AD pathology, compared with none of the 6 agrammatic patients, 3 of 5 of the “mixed” patients, and the single semantic patient. It is important to recognize that classification of PPA phenotypes generally depends on syndromic characterization, and overlap between syndromes is frequent, particularly with disease evolution (e.g., LPA overlaps both with PNFA and SD). It is unclear whether older series of PPA cases included patients that would now be described as having LPA, e.g., in Alladi et al. (2007) many of the patients with PNFA were diagnosed before the initial description of LPA. In that study, 5 of 7 patients with a mixed aphasia (including LPA) patients had AD pathology, compared with 2 of 20 with SD and 12 of 26 with PNFA. Improved understanding of the specific disease phenotypes has refined clinicopathological correlations in PPA, e.g., patients with motor speech deficits (e.g., apraxia of speech) appear to show an association with FTLD-tau rather than AD pathology (Josephs et al., 2006). For the clinical syndrome of SD there is an association chiefly with FTLD-TDP rather than AD pathology (Alladi et al., 2007; Snowden et al., 2007). The SD syndrome underpinned by AD may be associated with asymmetrical temporal lobe atrophy focused on the left hippocampus and superior temporal lobe, rather than the temporal pole and anteroinferior temporal lobe as in classical SD caused by FTLD-TDP pathology (Chan et al., 2001; Pereira et al., 2009; Rohrer et al., 2009). More marked superior temporal lobe atrophy has been associated with LPA in other studies (Gorno-Tempini et al., 2004, 2008).

An outstanding neurobiological question concerns the overlap of LPA/atypical language-presentation AD with typical amnestic AD (and with other atypical variants of AD such as posterior cortical atrophy). Neuropsychologically, there are few data to compare amnestic-onset AD with atypical language variants but studies of language impairment in typical AD have shown that patients can be logopenic with an early anomia, and that phonological and semantic impairments also occur (Adlam et al., 2006; Blair et al., 2007; Chertkow et al., 2008; Garrard et al., 2001; Harasty et al., 1999, 2001; Peters et al., 2009; Taler and Phillips, 2008). Motor speech impairment (apraxia of speech) has been reported only rarely in association with AD (Gerstner et al., 2007). From an anatomical perspective, LPA is associated with asymmetrical atrophy compared with the relatively symmetrical atrophy of amnestic AD (Gorno-Tempini et al., 2004). However, certain key areas of atrophy or cortical thinning are implicated in both LPA-AD and typical AD, i.e., the temporo-parietal junction, the precuneus, posterior cingulate, and the medial temporal lobe (Scahill et al., 2002). One recent study has shown an overlap of patterns of atrophy in these areas in early onset amnestic AD, posterior cortical atrophy, and LPA (Migliaccio et al., 2009). The present study has certain limitations, including relatively small patient numbers, retrospective ascertainment, and most importantly, lack of uniform histopathological confirmation. Taking these caveats into account, the present evidence in conjunction with previous work suggests that the LPA syndrome might be regarded, very broadly, as a “uni-hemispheric” presentation of AD. Further detailed longitudinal prospective studies comparing amnestic and language presentations of AD are needed to elucidate the pathophysiological mechanisms that instigate and sustain neuropsychological and anatomical asymmetry.

## Disclosure statement

Dr. Rohrer has received research support from Brain (Exit Scholarship). Dr. Rossor serves on a scientific advisory board for Elan Corporation and Wyeth; serves as Editor-in-Chief of the Journal of Neurology, Neurosurgery and Psychiatry, and on the editorial boards of Practical Neurology, Dementia and Geriatric Cognitive Disorders, Neurodegenerative Diseases, and the British Medical Journal; receives royalties from publishing Brain's Diseases of the Nervous System (11th Ed.), Oxford University Press (2001), and Brain's Diseases of the Nervous System (12th Ed.), Oxford University Press (2009); and receives research support from the Department of Health and the Alzheimer's Research Trust. Dr. Warren has received research support from the Wellcome Trust (Intermediate Clinical Fellowship).

Ethics approval was obtained from the local ethics committee at the National Hospital for Neurology and Neurosurgery, London, UK. Written research consent was obtained from all patients participating in the study.

## Figures and Tables

**Table 1:** Demographic, symptom, and pathology data

Patient	Gender	Age at onset	Total duration	First symptoms	Other linguistic symptoms	Neurological and behavioural symptoms	CSF	Tissue pathology
AD-PPA1	M	59	9.3	Word-finding difficulty	Phonemic errors, later comprehension problems	Myoclonus and seizures	N/A	Braak VI, CERAD frequent plaques, Reagan high
AD-PPA2	F	54	8.1	Word-finding difficulty	Phonemic errors, sentence repetition impairment	Seizures	N/A	Braak VI, CERAD frequent plaques, Reagan high. Mild cerebral amyloid angiopathy
AD-PPA3	M	50	6.3	Word-finding difficulty	Phonemic errors	Myoclonus	N/A	Severe pathology – frequent plaques and tangles. Extensive amyloid angiopathy
AD-PPA4	M	62	5.2	Return of childhood stutter	Word-finding difficulty, phonemic and jargon errors	Nil other noted	N/A	Braak VI, CERAD frequent plaques, Reagan high. Severe cerebral amyloid angiopathy
AD-PPA5	F	66	9.7	Word-finding difficulty	Phonemic errors, sentence repetition impairment	Nil other noted	N/A	Braak VI, CERAD frequent plaques, Reagan high. Severe cerebral amyloid angiopathy
AD-PPA6	M	50	7.2	Word-finding difficulty	Phonemic and jargon errors, later comprehension problems	Later aggressive behaviour	N/A	Braak VI, CERAD frequent plaques, Reagan high
AD-PPA7	M	54	8.9	Word-finding difficulty	Phonemic errors	Later axial rigidity Later aggressive behaviour	N/A	Braak VI, CERAD frequent plaques, Reagan high
AD-PPA8	F	50	N/A	Word-finding difficulty	Phonemic errors	Anxiety	N/A	Cerebral biopsy: Frequent plaques and tangles
AD-PPA9	M	48	N/A	Word-finding difficulty	Phonemic errors	Nil other notes	N/A	Cerebral biopsy: Frequent plaques and tangles
AD-PPA10	M	60	N/A	Word-finding difficulty	Phonemic errors	Later anxiety, irritability and disinhibition	tau > 1200 ng/L; Aβ42 195 ng/L	N/A
AD-PPA11	M	53	N/A	Word-finding difficulty	Phonemic errors	Irritability	tau 1146 ng/L, Aβ42 250 ng/L	N/A
AD-PPA12	F	63	N/A	Word-finding difficulty	Phonemic errors	Anxiety and apathy	tau 1124 ng/L, Aβ42 299 ng/L	N/A
AD-PPA13	M	59	N/A	Word-finding difficulty	Phonemic errors	Irritability, restlessness and agitation	tau 986 ng/L, Aβ42 138 ng/L	N/A
AD-PPA14	M	58	N/A	Word-finding difficulty	Phonemic and jargon errors, later comprehension problems	Anxiety	tau 986 ng/L, Aβ42 130 ng/L	N/A

Cases shown in bold represent patients with CSF data consistent with AD, other cases are pathologically confirmed cases. Key: AD, Alzheimer's disease; CERAD, Consortium to Establish a Registry for Alzheimer's Disease; CSF, cerebrospinal fluid; F, female; M, male; PPA, primary progressive aphasia.

**Table 2:** Neuropsychological data

Patient	Duration at assessment	MMSE	VIQ a	PIQ^a^	Naming	Single word comprehension	Digit Span forwards	Verbal memory	Visual memory	Reading	Limb praxis	Calculation	Visuospatial skills	Executive function
AD-PPA1	4.1	17	61	74	−	−	−	+	+	− (phon)	−	−	+	+
AD-PPA2	3.7	26	83	99	+	+	−	−	+	NT	−	−	+	−
AD-PPA3	2.8	17	66	64	+	+	−	−	+	NT	−	−	+	−
AD-PPA4	3.2	4	Unable	Unable	−	−	−	−	−	− (phon)	−	−	−	−
AD-PPA5	4.0	21	84	68	−	+	+	−	−	+	−	−	+	−
AD-PPA6	3.1	27	79	122	+	+	−	+	+	− (phon)	NT	+	+	+
AD-PPA7	3.1	20	70	107	−	+	−	+	+	−	NT	−	+	+
AD-PPA8	2.3	18	61	79	+	+	−	−	+	+	−	+	+	−
AD-PPA9	2.3	NT	85	91	+	+	−	−	+	−	−	+	+	−
AD-PPA10	5.4	8	NT	NT	−	−	−	NT	+	− (phon)	−	−	+	−
AD-PPA11	3.0	21	79	80	+	+	+	−	−	+	−	−	+	−
AD-PPA12	3.1	17	81	84	−	+	+	−	−	− (phon)	−	−	+	−
AD-PPA13	3.9	16	62	77	−	−	−	NT	+	− (phon)	−	−	+	−
AD-PPA14	4.8	8	NT	NT	−	−	−	NT	−	− (phon)	−	−	+	−

+ Represents intact function, − represents impaired function, i.e., a score below the 5th percentile on testing; for reading score (phon) represents the presence of a phonological dyslexia. Verbal and visual memory were tested with the Warrington Recognition Memory Test for Words and Faces, naming with the Graded Naming Test, single word comprehension with the WAIS-R vocabulary subtest or Warrington synonyms test, reading with the National Adult Reading Test or Schonell reading test, visuospatial skills with the Visual Object and Space Perception battery, digit span with the WAIS-R digit span subtest, calculation with the Graded Difficulty Calculation Test (GDCT), and executive function with the Weigl or Wisconsin Modified Card Sorting Tasks or Stroop task. Key: AD, Alzheimer's disease; MMSE, Mini Mental State Examination; NT, not tested; PIQ, Performance Intelligence Quotient; PPA, primary progressive aphasia; VIQ, Verbal Intelligence Quotient; WAIS-R, Wechsler Adult Intelligence Scale-Revised.

a Scores taken from the WAIS-R.

**Table 3:** Volumetric MRI data

	Controls	AD-PPA
Number of subjects	23	14
Duration of disease at scan, years	N/A	4.1 (1.0)
Age at scan, years	63.5 (7.3)	60.2 (6.2)
Brain volume, mL	1160.1 (96.5)	1083.7 (109.1)^a^
Left hemisphere volume, mL	570.9 (46.7)	526.4 (57.0)^a^
Right hemisphere volume, mL	571.3 (46.9)	547.9 (50.6)
Left/right hemisphere ratio	1.00 (0.01)	0.96 (0.03)^a^

Mean (standard deviation) values are shown. AD, Alzheimer's disease; MRI, magnetic resonance imaging; N/A, not applicable; PPA, primary progressive aphasia.

a *p* < 0.05 AD-PPA significantly worse than controls.

**Table 4:** Previously reported series of patients with a primary progressive aphasia and Alzheimer pathology

Series	Cases considered	Pathologically confirmed AD, *n*	PPA diagnosis	Male, %	Age at onset	Duration	Age at death
Migliaccio et al., 2009^a^	Only LPA cases	1 and 4 with positive PIB scan	5 LPA	NA	NA	NA	NA
Pereira et al., 2009^b^	Only SD cases	3	3 SD	66.7	NA	NA	NA
Rabinovici et al., 2008^a^	All PPA cases	0 but 6 with positive PIB-PET scan	4 LPA, 1 SD, 1 PNFA	NA	NA	NA	NA
Gorno-Tempini et al., 2008^a^	Only LPA cases	0 but 4 with positive PIB-PET scan	4 LPA	25.0	NA	NA	NA
Mesulam et al., 2008	All PPA cases	11	7 LPA, 1 SD, 3 “mixed”	63.6	61.8 (10.8)	NA	73.2 (7.0)
Josephs et al., 2008	All PPA cases	5	5 “Fluent aphasia” (“1 or 2… may meet criteria for logopenic PPA”)	60.0	69 (12)	NA	77 (13)
Alladi et al., 2007^b^	All PPA cases	19	12 PNFA, 2 SD, 5 “mixed” (“mixed” cases include 3 LPA, 2 atypical SD with phonological deficits)	NA	65.7 (8.1)	7.4 (2.9)	NA
Knibb et al., 2006^b^	All PPA cases	12	7 “Nonfluent”, 5 “fluent”	NA	NA	NA	NA
Kertesz et al., 2005	PNFA and LPA cases	8	8 PPA	NA	NA	NA	NA

Mean (standard deviation) values are shown. AD, Alzheimer's disease; LPA, logopenic/phonological aphasia; NA, not available; SD, semantic dementia; PET, positron emission tomography; PIB, ; PNFA, progressive nonfluent aphasia; PPA, primary progressive aphasia.

a From same research group and cases may overlap in different series.

b From same research group and cases may overlap in different series. Note earlier series which include AD-PPA cases are Davies et al. (2005); Croot et al. (2000), and Galton et al. (2000).
